# Population-level effectiveness of alternative approaches to preventing mental disorders in adolescents and young adults

**DOI:** 10.1038/s41598-023-47322-2

**Published:** 2023-11-15

**Authors:** Adam Skinner, Jo-An Occhipinti, Yun Ju Christine Song, Ian B. Hickie

**Affiliations:** 1 Brain and Mind Centre, Faculty of Medicine and Health, University of Sydney, Sydney, Australia; 2 Computer Simulation and Advanced Research Technologies (CSART), Sydney, Australia

**Keywords:** Psychiatric disorders, Public health, Risk factors, Applied mathematics

## Abstract

Preventive interventions that are effective in reducing the incidence of mental disorders in adolescence and early adulthood may impact substantially on lifetime economic, educational, and health outcomes; however, relatively few studies have examined the capacity of alternative approaches to preventing youth mental disorders (specifically, universal, selective, and indicated prevention) to reduce disorder incidence at a population level. Using a dynamic model of the onset of non-specific, relatively mild symptoms and progression to more severe disease, we show that: (1) indicated preventive interventions, targeting adolescents and young adults experiencing subthreshold symptoms, may often be more effective in reducing mental disorder prevalence than universal interventions delivered to the general population (contrary to the widely accepted view that a ‘high risk’ prevention strategy, focussing on those individuals with the greatest risk of developing a disorder, will generally be less effective than a whole-population strategy); and (2) the ability of selective preventive interventions (targeting vulnerable, asymptomatic youth) to alter the prevalence of mental disorders is severely restricted by an inverse relationship between the prevalence of significant risk factors for mental illness and the relative risk of developing symptoms.

## Introduction

Mental disorders are a principal cause of disability globally, directly affecting an estimated 12.6% of the world population and accounting for 14.6% of years lived with disability (ranked 2nd, after musculoskeletal disorders)^1^. Nearly two-thirds of people with a mental disorder (62.5%) first develop symptoms as adolescents or young adults (before the age of 25), with a peak age of onset of 14.5 years (median age of onset 18 years)^2^. Adolescence and early adulthood are critical periods for establishing the social, cultural, emotional, and educational resources underpinning both the successful adoption of adult roles and responsibilities (economic participation, civic engagement, parenthood, etc.) and the maintenance of health and wellbeing into middle and late adulthood^3^. Prospective analyses indicate that the development of a serious mental disorder during this pivotal stage of life is a significant predictor of persistent, adverse socioeconomic and health outcomes, including economic non-participation, unemployment, low income, welfare dependency, low educational attainment (no post-secondary qualification), and poor physical health^4–6^. Nevertheless, available evidence also indicates that relatively mild mental disorders originating in adolescence frequently do not persist into early adulthood^7^, so that effective, youth-focussed interventions designed to reduce the risk of symptom onset or prevent progression from relatively mild to more severe mental health problems have the potential to impact substantially on longer-term economic, educational, and health outcomes.

Preventive interventions for mental disorders can be divided into three principal types according to the average risk of developing a disorder among individuals in the target population^8,9^, namely (in order of increasing risk): (1) universal preventive interventions, which are considered appropriate for a general target population defined on the basis of some criterion unrelated to the risk of mental illness (e.g., mental health awareness programs for secondary school students); (2) selective preventive interventions, targeting asymptomatic individuals exposed to one or more biological, psychological, or socioeconomic risk factors that significantly increase their risk of developing a mental disorder (e.g., preventive psychological interventions for children of parents with major depressive disorders); and (3) indicated preventive interventions, which aim to prevent progression to full-threshold disorders (clinical stages 2 and above)^10,11^ in individuals presenting with non-specific, relatively mild symptoms (early stage 1), typically through the provision of psychological and social support. Although many studies have examined the individual-level effectiveness of particular universal, selective, and indicated interventions in preventing mental disorders in adolescents and young adults^9,12^, the potential impacts of these alternative approaches to prevention on population mental health (which will depend on both the reach and individual-level efficacy of an intervention) have received considerably less attention. This paper presents a dynamic modelling analysis of the general conditions under which each type of intervention (i.e., universal, selective, and indicated) would be expected to significantly reduce the incidence of mental disorders in adolescence and early adulthood, providing a framework for assessing the potential population-level impacts of specific preventive interventions and, more broadly, for guiding the development of effective prevention strategies.

## Results

### Model structure and assumptions

**Figure 1:**
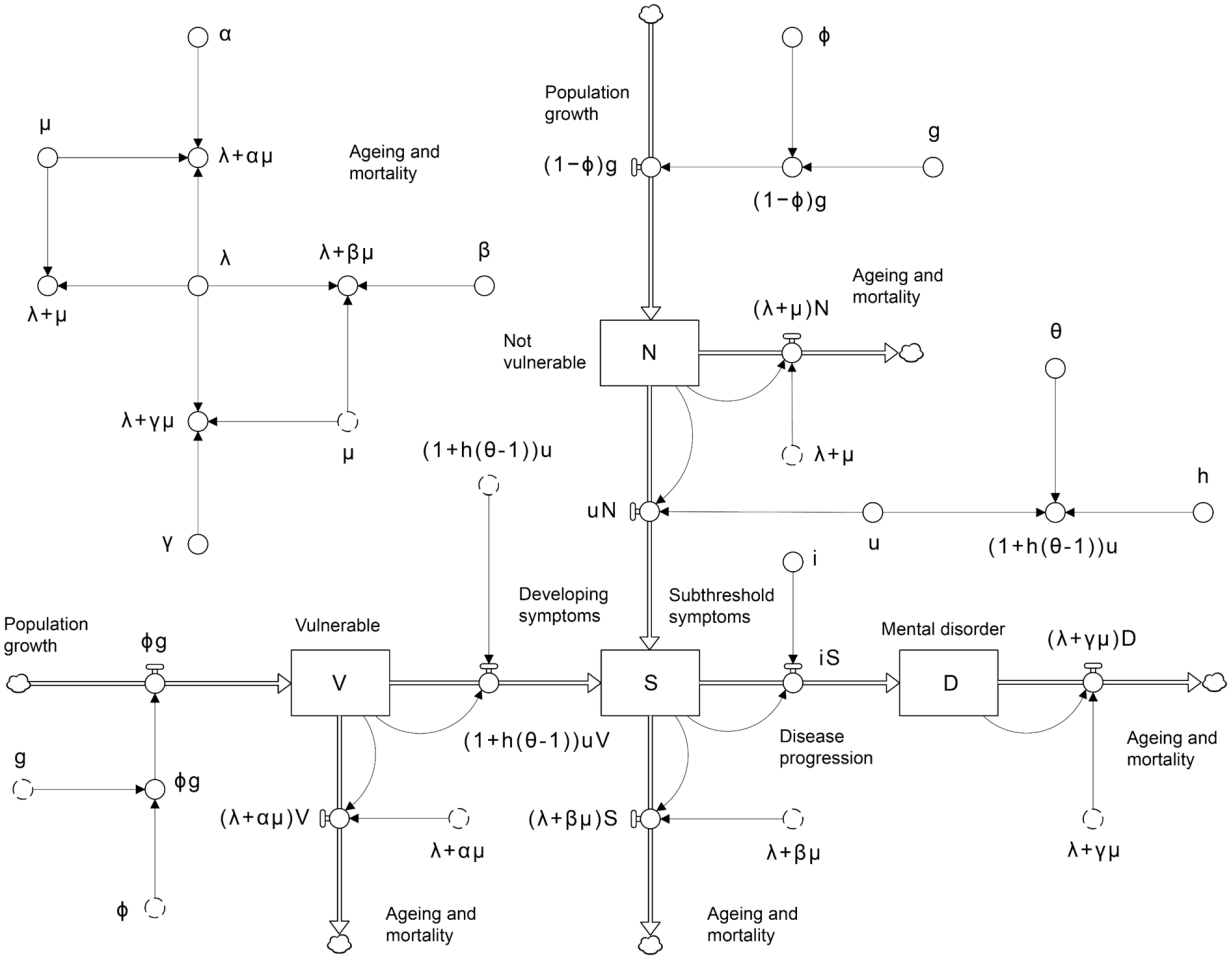
Dynamic model used for the analysis. Notation is defined in the Results section and Table 1. Stocks (or compartments, state variables) are shown as boxes, flows as pipes with taps, causal connections (or mathematical dependencies) as arrows, and sources and sinks as clouds (see refs.^13^, ^14^). Symbols with dashed outlines are copies (or ‘ghosts’) of the corresponding symbols with solid outlines.

The dynamic model developed for the analyses (Fig. 1) comprises four stocks, or compartments, corresponding to adolescents and young adults (youth, aged 12–24 years) who are asymptomatic and have a relatively low risk of developing a mental disorder (not vulnerable, labelled $N$); are asymptomatic, but are currently exposed to one or more risk-modifying factors that significantly increase their risk of developing symptoms (vulnerable, $V$); are experiencing non-specific, relatively mild symptoms associated with minimal functional impairment (subthreshold symptoms, $S$); and who have full-threshold mental disorders, characterised by more severe and persistent symptoms and significantly impaired social, educational, and occupational functioning (mental disorder, $D$). Adolescents flow into the stocks $N$ and $V$ at rates equal to $\left(1-\phi \right)g$ and $\phi g$, respectively, where $g$ is the yearly increase in the youth population due to ageing (i.e., adolescents aged 11 years turning 12 years) and $\phi$ is the proportion of adolescents turning 12 years who are vulnerable (migration is assumed to be negligible). The value of the parameter $\phi$ (and also the symptom onset hazard ratio $\theta$; see below) will depend on the specific risk factors considered relevant in defining the vulnerable youth population, which will usually correspond to the target population for one or more selective preventive interventions (e.g., for selective interventions aimed at preventing mental disorders among children of parents with major depressive disorders, $\phi$ would equal the proportion of adolescents exposed to parental depression). For simplicity, the risk of developing symptoms is assumed to remain stable throughout adolescence and early adulthood, so there are no flows connecting the vulnerable and non-vulnerable stocks.

Non-vulnerable and vulnerable youth develop symptoms at rates (per year) equal to $uN$ and $\left[1+h\left(\theta -1\right)\right]uV$, respectively, where $u$ is the per capita symptom onset rate for the non-vulnerable population, $h$ is the proportion of vulnerable adolescents and young adults at increased risk of developing symptoms (equal to 1 prior to implementing selective preventive interventions, which act to reduce $h$), and the hazard ratio $\theta$, equal to the per capita symptom onset rate for the vulnerable population divided by $u$, is assumed to be greater than 1 (since the risk of symptom onset is, by definition, higher for the vulnerable population than for the non-vulnerable population). Progression from subthreshold symptoms (corresponding to clinical stages 1a–b) to full-threshold mental disorders (stages 2–4) occurs at a per capita rate $i$, so that $iS$ adolescents and young adults develop full-threshold disorders per year. Mortality and ageing remove adolescents and young adults from the non-vulnerable, vulnerable, mildly symptomatic, and full-threshold disorder stocks at yearly rates equal to $\left(\lambda +\mu \right)N$, $\left(\lambda +\alpha \mu \right)V$, $\left(\lambda +\beta \mu \right)S$, and $\left(\lambda +\gamma \mu \right)D$, respectively, where $\lambda$ is the reciprocal of the time in years spent in the youth population (i.e., before turning 25 years), $\mu$ is the per capita mortality rate for the non-vulnerable population, and $\alpha$, $\beta$, and $\gamma$ are mortality hazard ratios (we assume $1\le \alpha <\beta <\gamma$, so that per capita mortality is highest for adolescents and young adults with full-threshold disorders, and higher for the mildly symptomatic population than for the vulnerable population).

### Model analysis

**Figure 2:**
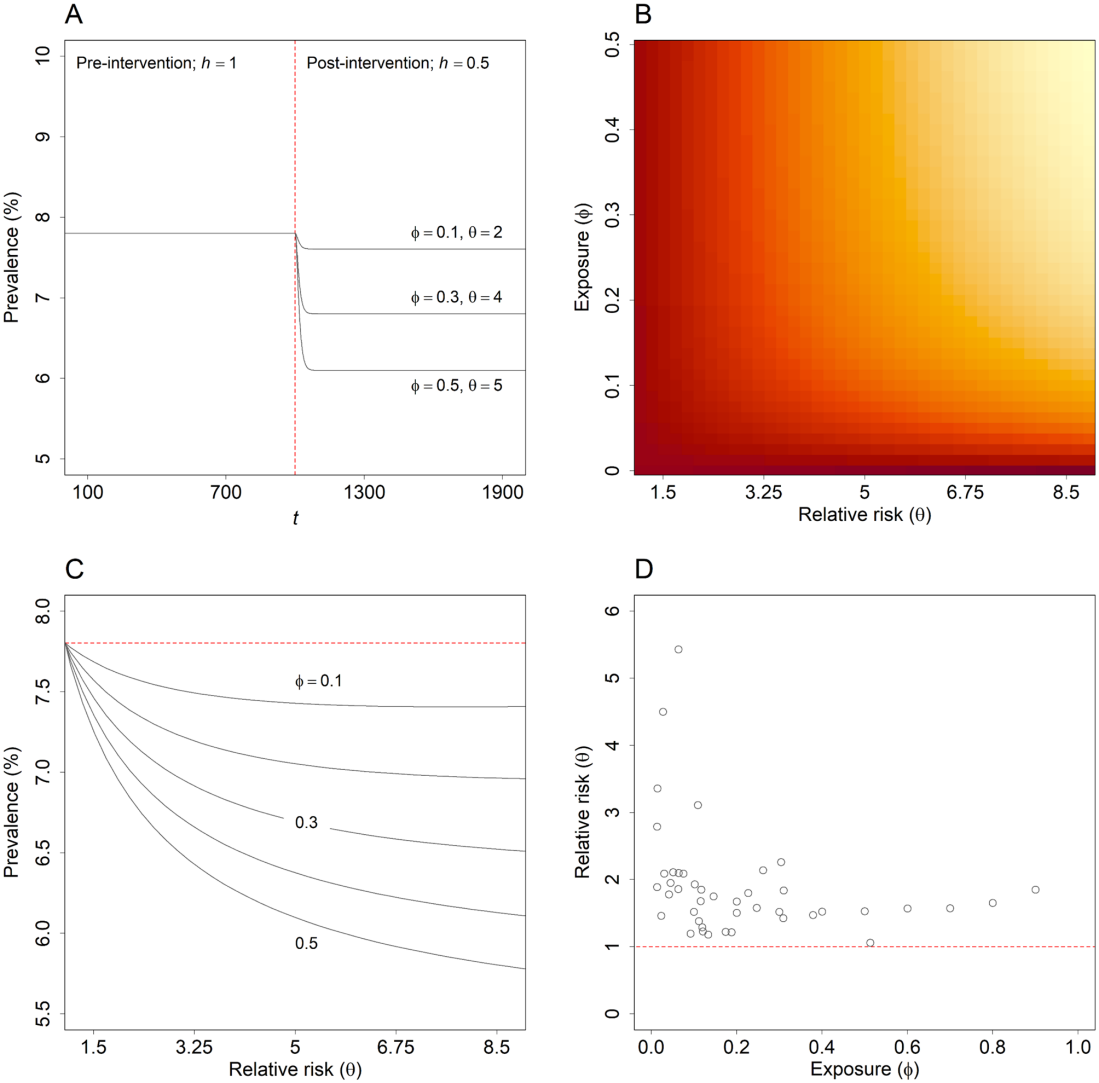
Effectiveness of selective preventive interventions and empirical estimates of $\phi$ and $\theta$. (**A**) Prevalence of full-threshold mental disorders under scenarios in which a selective intervention that halves the proportion of vulnerable adolescents and young adults at increased risk of developing symptoms ($h$ equal to 0.5) is introduced at time $t$ = 10^3^ and remains in place indefinitely. Results are presented for three sets of values for $\phi$ and $\theta$. (**B**–**C**) Post-intervention equilibrium prevalence of full-threshold mental disorders as a function of the parameters $\phi$ and $\theta$ (i.e., the proportion of adolescents turning 12 years exposed to one or more risk factors for mental illness and the relative risk of symptom onset, respectively). Dark red shading in panel B corresponds to higher equilibrium prevalence, pale yellow shading to lower equilibrium prevalence. In all cases, the equilibrium prevalence of full-threshold disorders is calculated assuming a value of 0.5 for $h$ (see Results section for further details). (**D**) Empirical estimates of $\phi$ and $\theta$ for a diverse selection of risk factors (see Supplementary Table S1 for details).

Panels A–C of Fig. 2 show the effect of an arbitrary selective intervention on the equilibrium prevalence of full-threshold disorders, $D/\left(N+V+S+D\right)$, as a function of the parameters $\phi$ and $\theta$ (equations for calculating the equilibrium values of the stocks $N$, $V$, $S$, and $D$ are derived in the Supplementary Information). Post-intervention equilibrium prevalence was calculated for each combination of values $\left(\phi ,\theta \right)$ assuming a value of 0.5 for $h$, so that the intervention halves the proportion of vulnerable adolescents and young adults at increased risk of developing subthreshold symptoms (see Fig. 2, panel A). The value of the per capita symptom onset rate for the non-vulnerable population, $u$, was specified such that the equilibrium prevalence of mental disorders in the absence of the intervention (i.e., with $h$ set to 1) is always equal to the proportion of Australian adolescents and young adults estimated to have had full-threshold disorders (clinical stages 2–4) in 2007 (7.80%, the most recent pre-COVID-19 estimate available)^11,15^. Accordingly, the equilibrium prevalence values calculated for different combinations of values $\left(\phi ,\theta \right)$ are directly comparable, in that the population-level intervention effects for two combinations of values yielding the same equilibrium prevalence are identical (the decrease in prevalence due to the intervention is the same in each case). As the proportion of adolescents entering the youth population who are vulnerable (i.e., the value of $\phi$) increases, the equilibrium prevalence of mental disorders declines, since the reach of the intervention, and therefore the absolute reduction in the number of adolescents and young adults at increased risk of developing symptoms, increases. Similarly, equilibrium disorder prevalence declines as the symptom onset hazard ratio for the vulnerable population increases, as higher values of $\theta$ permit a greater reduction in the risk of developing symptoms for each person receiving the intervention.

**Figure 3:**
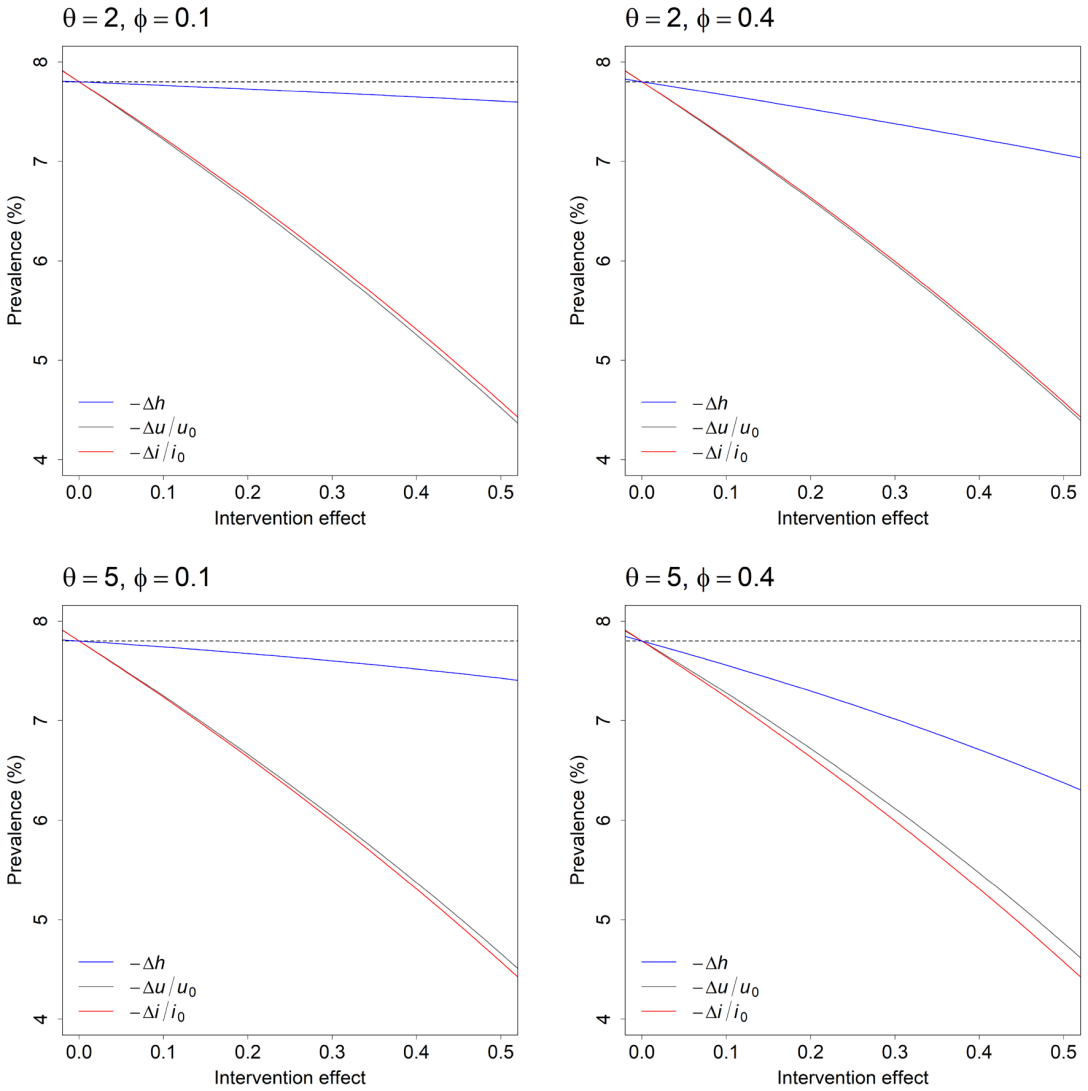
Population-level effectiveness of universal, selective, and indicated preventive interventions for youth mental disorders. Each panel presents the post-intervention equilibrium prevalence of full-threshold disorders for universal (grey), selective (blue), and indicated (red) preventive interventions with varying individual-level effects ($-\Delta u/{u}_{0}$, $-\Delta h$, and $-\Delta i/{i}_{0}$, respectively; see Results section for further details). Note that the population-level effectiveness of a selective preventive intervention is strongly dependent upon the parameters $\phi$ and $\theta$ (i.e., the proportion of adolescents turning 12 years exposed to one or more risk factors for mental illness and the relative risk of developing subthreshold symptoms, respectively), as well as the individual-level intervention effect $-\Delta h$.

Figure 3 compares the potential effects of hypothetical universal, selective, and indicated preventive interventions on the equilibrium prevalence of mental disorders under four alternative sets of values for the parameters $\phi$ and $\theta$. The horizontal axis in each panel is the individual-level intervention effect, equal to the proportional reduction in the per capita symptom onset rate $u$ for universal preventive interventions (i.e., $-\Delta u/{u}_{0}$, where ${u}_{0}$ is the pre-intervention value of $u$, and $\Delta u=u-{u}_{0}$), the proportional reduction in the per capita disease progression rate $i$ for indicated preventive interventions ($-\Delta i/{i}_{0}$), and the absolute reduction in $h$ for selective preventive interventions ($-\Delta h$, which is also the proportional reduction). For any specified individual-level effect (e.g., 0.2, corresponding to a 20% decrease in the values of $u$, $i$, and $h$), universal and indicated preventive interventions are nearly equally effective in reducing the equilibrium prevalence of mental disorders, and generally much more effective than selective preventive interventions, at least for the specific values of $\phi$ and $\theta$ considered here. Nevertheless, the individual-level effects of alternative interventions may differ significantly, so that each type of intervention could, in principle, have a greater population-level effect than the other intervention types. An indicated intervention that is moderately effective in preventing disease progression, for example, will be considerably more effective in reducing equilibrium disorder prevalence than a universal intervention that reduces the per capita symptom onset rate only marginally, while selective interventions that substantially reduce the proportion of vulnerable adolescents and young adults at increased risk of developing symptoms may reduce the equilibrium prevalence of disorders to a greater extent than universal and indicated interventions with lower individual-level effects, provided that the values of $\phi$ and $\theta$ are reasonably high.

### Real-world intervention impacts

While the results presented in Fig. 3 enable us to identify general conditions under which universal, selective, and indicated approaches to prevention will significantly reduce the prevalence of mental disorders in an adolescent and young adult population, they provide no indication of the range of conditions that could reasonably be expected in real-world settings (specifically, the individual-level effects of available preventive interventions and the values of $\phi$ and $\theta$). Empirical estimates of $\phi$ and $\theta$ for a diverse selection of risk-modifying factors affecting mental disorder incidence in adolescence and early adulthood, including childhood trauma (physical or sexual abuse, death of a parent, etc.), parental mental disorders, socioeconomic disadvantage, ethnicity, and physical health and health-related behaviours are presented in panel D of Fig. 2 (see Supplementary Table S1 for details). Most estimates of the hazard ratio $\theta$ are less than 2 (74.42%) and estimates higher than 3 are associated with relatively low values of $\phi$ (*c*. 0.1 or less), so that the population-level effects of selective preventive interventions will typically be restricted to those indicated in the upper panels and lower left panel of Fig. 3. Meta-analyses of randomised controlled trials evaluating the efficacy of psychological and educational preventive interventions for high-prevalence mental disorders indicate that individual-level intervention effects are on the order of 0.21 for universal preventive interventions (0.10 for depression, 0.33 for anxiety), 0.44 for selective preventive interventions (0.28 for depression, 0.60 for anxiety), and 0.31 for indicated preventive interventions (0.24 for depression, 0.39 for anxiety)^18,19^. Assuming values of 0.4 for $\phi$ and 2 for $\theta$ (see Fig. 3, upper right panel), universal, selective, and indicated preventive interventions with these individual-level effects (0.21, 0.44, and 0.31, respectively) reduce the equilibrium prevalence of full-threshold disorders in our model population from 7.80% to 6.55%, 7.17%, and 5.93%, respectively.

## Discussion

The analysis presented above provides supporting evidence for three principal conclusions about the capacity of preventive interventions to reduce mental disorder incidence in adolescence and early adulthood that have significant implications for public health policy and services planning decisions. Firstly, an inverse relationship between empirical values of $\phi$ and $\theta$ (see panel D of Fig. 2) severely restricts the potential effectiveness of selective interventions in reducing the incidence (and therefore prevalence) of youth mental disorders at a population level. Selective preventive interventions targeting adolescents and young adults at very high risk of developing subthreshold symptoms (e.g., survivors of childhood physical or sexual abuse) will usually be delivered to only a small proportion of the total population with elevated risk (for any reason), while adolescents and young adults exposed to more widely occurring risk factors (parental separation, residential instability, etc.) generally have only a moderately increased risk of developing symptoms (so the potential for reducing individual-level risk is relatively low). Secondly, universal and indicated interventions that are equally effective at an individual level are expected to produce similar reductions in disorder incidence at a population level (i.e., assuming the intervention effects $-\Delta u/{u}_{0}$ and $-\Delta i/{i}_{0}$ are equal). Nevertheless, because the individual-level effectiveness of indicated preventive interventions is generally higher than that of universal interventions^18–20^, indicated prevention will typically produce greater population-level effects than universal and selective approaches (at least for available interventions). And thirdly, substantial increases in the population-level effectiveness of multi-component prevention programs may be achieved via increases in the individual-level effectiveness of universal and indicated preventive interventions or, to a lesser extent, by improving risk prediction models (which would tend to increase $\theta$ relative to $\phi$); however, any effect of increases in the individual-level effectiveness of selective preventive interventions is likely to be limited (Fig. 3).

According to Rose’s *Strategy of preventive medicine*^21^, a substantial proportion of incident cases of many (perhaps most) disorders will occur among individuals at relatively low risk of developing symptoms simply because these individuals comprise most of the population, so that approaches to prevention focussing on the smaller number of individuals at very high risk (the ‘high-risk’ strategy) may often be less effective in reducing disorder prevalence than approaches aimed at decreasing the mean level of risk for the population as a whole (the population strategy). The results presented here for universal and selective preventive interventions clearly demonstrate the potential for whole-population approaches (i.e., universal prevention) to deliver greater population-level effects than targeted approaches; where the proportion of adolescents and young adults at significantly increased risk of developing symptoms is low (e.g., where $\phi$ is equal to 0.1; left panels of Fig. 3), universal interventions that are at least moderately effective at the individual level ($-\Delta u/{u}_{0}$ equal to 0.2 or higher) are generally considerably more effective in reducing the prevalence of full-threshold mental disorders than selective interventions. Nevertheless, our results also provide evidence that indicated preventive interventions, targeting adolescents and young adults experiencing subthreshold symptoms, will typically yield population-level effects comparable to (and often greater than) those expected for universal approaches. Although indicated preventive interventions are delivered to a small target population of high-risk individuals (and therefore align with Rose’s ‘high-risk’ strategy), this target population includes everyone who would develop a full-threshold disorder in the absence of the intervention (adolescents and young adults are assumed to always develop non-specific, relatively mild symptoms before progressing to more severe, full-threshold disorders, consistent with clinical stage models)^10,11^. As a result, indicated interventions, unlike selective interventions, have the same, unrestricted capacity to prevent disorder onset as universal interventions delivered to the general population (in both cases, nobody who would develop a mental disorder in the absence of the intervention is precluded from receiving the intervention).

Despite the focus of our analysis on evaluating and comparing the population-level impacts of single preventive interventions, we assume that any comprehensive prevention strategy capable of substantially reducing not only the prevalence of more severe mental disorders, but also the incidence of relatively mild, subthreshold symptoms, demand for (and therefore pressure on) mental health services, and social and economic inequalities in disorder prevalence, will necessarily include a mix of evidenced-based universal, selective, and indicated interventions^8,9,22^. The effectiveness of a multifaceted prevention program in reducing the burden of youth mental disorders will depend principally on the individual-level effectiveness of its component universal and indicated interventions and, to a more limited extent, on the reliability with which the risk of symptom onset can be predicted for vulnerable adolescents and young adults (since this determines the values of $\theta$ and $\phi$; Fig. 3 and Supplementary Fig. S1). Equilibrium disorder prevalence declines rapidly as $-\Delta u/{u}_{0}$ and $-\Delta i/{i}_{0}$ increase (Fig. 3), so that substantial reductions in the occurrence of mental illness are expected as the ability of universal and indicated preventive interventions to restrict per capita rates of symptom onset and disease progression improves. Potentially significant increases in the effectiveness of multi-component prevention programs may therefore be achieved by increasing investment in interventions aimed at promoting early engagement with services, improving treatment for adolescents and young adults experiencing subthreshold symptoms (e.g., via the routine use of health information technologies to deliver highly personalised and measurement-based care)^23^, and reducing exposure to widespread social and economic risk factors for poor mental health (e.g., underemployment and insecure employment, financial hardship, income inequality, gender-based violence, and social disconnection)^24^.

There are several limitations of the analysis presented here that should be pointed out. Firstly, our model only captures aggregate (i.e., population-level) dynamics, and so effectively disregards potentially significant heterogeneity in individual-level factors that are expected to influence the incidence of subthreshold symptoms, engagement with services, progression to more severe disorders, and responses to specific preventive interventions (principal diagnosis, comorbidities, age, gender, etc.^8^). Analyses of more complex dynamic models (particularly agent-based models)^25,26^ are needed to determine the degree to which the individual-level variability of these factors may affect our conclusions. Secondly, the values assumed for the parameters $u$ and $i$ (i.e., the per capita rate of symptom onset for the non-vulnerable population and the per capita disease progression rate, respectively) were derived from data on the prevalence of mental disorders among Australian adolescents and young adults and are not necessarily generalisable to other populations (see Results section and Table 1). Nevertheless, analyses using alternative prevalence estimates from the World Health Organization’s World Mental Health Surveys (see Supplementary Figs. S2–S4)^27^ yield results qualitatively similar to those presented in Fig. 3, indicating that our conclusions are not dependent upon the particular parameter values (for $u$ and $i$) specified in our analysis. And finally, although the prevalence of full-threshold disorders is expected to converge to a stable equilibrium value over time (see Supplementary Information; Fig. 2, panel A), convergence can be relatively slow, so that the full population-level effect of a preventive intervention may only be realised over an extended period. As a consequence, interventions that have similar impacts on the equilibrium prevalence of youth mental disorders will not necessarily produce similar reductions in disorder prevalence over shorter time intervals (Supplementary Fig. S5).

**Table 1:** Parameter values assumed in the model analysis.

Parameter	Symbol	Value	Reference(s)
Initial population	*P*_*0*_	1,000,000	
Population increase per year	*g*	–	
Proportion of adolescents turning 12 at increased risk of developing symptoms	*ϕ*	–	
Per capita symptom onset rate for non-vulnerable population	*u*	–	
Proportion of vulnerable population at increased risk of developing symptoms	*h*	1 (pre-intervention)	
Per capita disease progression rate	*i*	0.03251	^11^, ^15^
Symptom onset hazard ratio for vulnerable population	*θ*	–	
Ageing rate (reciprocal of the time in years spent in the youth population)	*λ*	0.07692	
Per capita mortality rate for non-vulnerable population	*μ*	0.0003343	^16^
Mortality hazard ratio for vulnerable population	*α*	1	
Mortality hazard ratio for population with subthreshold symptoms	*β*	1.2	^17^
Mortality hazard ratio for population with full-threshold mental disorders	*γ*	1.673	^17^

Values of $\phi$ and $\theta$ were varied as part of the analysis (see Figs. 2 and 3), $u$ and $i$ were set such that the equilibrium proportions of adolescents and young adults with subthreshold symptoms and full-threshold disorders are equal to empirical estimates for Australia (18.6% and 7.80%, respectively)^11,15^, and $g$ was set to produce a constant population of 1 million.

In conclusion, the dynamic modelling analysis presented here indicates that the ability of a multifaceted prevention program to reduce the incidence of youth mental disorders will depend primarily on the individual-level efficacy of its component universal and indicated interventions (see Fig. 3 and Supplementary Fig. S1). Significantly, our modelling demonstrates that universal and indicated preventive interventions that are equally effective at an individual level, producing identical proportional reductions in per capita rates of symptom onset and progression to more severe disease, respectively, will have similar effects on disorder incidence at a population level (contrary to the expectation that a ‘high-risk’ prevention strategy will generally be less effective than a whole-population strategy)^21^. At the same time, evidence from randomised controlled trials indicates that targeted preventive interventions for high-prevalence mental disorders are typically more effective (i.e., at an individual level) than universal preventive interventions^18–20^, so that indicated prevention may often produce greater population-level effects than universal prevention, at least for currently available interventions. An inverse relationship between the prevalence of significant risk factors for mental illness and the relative risk of developing subthreshold symptoms (values of $\phi$ and $\theta$, respectively) severely restricts the capacity of selective preventive interventions to alter the incidence of youth mental disorders at a population level; however, selective prevention may contribute significantly to reducing substantial social and economic inequalities in disorder prevalence, and so may be considered an integral part of any comprehensive prevention strategy aiming not only to improve population mental health, but also to provide vulnerable adolescents and young adults with the greatest possible chance of escaping lifelong disadvantage.

## Methods

### Model analysis

Population-level effects for alternative preventive interventions were assessed by determining the equilibrium prevalence of full-threshold mental disorders under scenarios in which a focal intervention with constant individual-level efficacy is introduced into the population and remains in place indefinitely (see panel A of Fig. 2). Equations for calculating the equilibrium values of the stocks $N$, $V$, $S$, and $D$, and evaluating (local) equilibrium stability are derived in the Supplementary Information. At the individual level, the effects of universal and indicated preventive interventions were modelled via proportional reductions in the per capita symptom onset rate $u$ (reducing the incidence of symptoms for both the non-vulnerable and vulnerable populations; Fig. 1) and the per capita disease progression rate $i$, respectively. For selective interventions, we modelled individual-level intervention effects as a decrease in the proportion of vulnerable adolescents and young adults at increased risk of developing symptoms (or, equivalently, a decrease in the mean level of risk for the vulnerable population as a whole), achieved via a reduction in the parameter $h$ (assumed to equal 1 where no selective interventions have been implemented). In calculating (post-intervention) equilibrium disorder prevalence, we specified the pre-intervention value of the per capita rate $u$ such that the equilibrium prevalence of mental disorders prior to introducing any preventive interventions was always equal to the proportion of Australian adolescents and young adults estimated to have had full-threshold disorders in 2007 (7.80%; values specified for the remaining model parameters are presented in Table 1). Post-intervention equilibrium prevalence values calculated for alternative intervention scenarios are therefore directly comparable, in that the population-level intervention effects for two scenarios yielding the same equilibrium prevalence values are identical (both the absolute decrease and relative decrease in prevalence due to the intervention are the same in each case).

### Supplementary Information

Supplementary Information.

## Data Availability

Details of all data sources used for the analyses are provided in Table 1 of the paper and the Supplementary Information.
