# Supplementary material for: Population-level effectiveness of alternative approaches to preventing mental disorders in adolescents and young adults
Source: Sci Rep. 2023 Nov 15;13:19982. doi: 10.1038/s41598-023-47322-2 (PMC10652005; doi:10.1038/s41598-023-47322-2)
Supplement: Supplementary file 1 — Supplementary Information. [file 41598_2023_47322_MOESM1_ESM.pdf]

Supplementary information. Population-level effectiveness of alternative approaches to preventing mental disorders in adolescents and young adults

Adam Skinner[1,*], Jo-An Occhipinti[1,2], Yun Ju Christine Song[1], Ian B. Hickie[1]

[1] Brain and Mind Centre, Faculty of Medicine and Health, University of Sydney, Sydney, Australia; Mental Wealth Initiative, University of Sydney, Sydney, Australia; [2] Computer Simulation and Advanced Research Technologies (CSART), Sydney, Australia

[*] Corresponding author. Email: adam.skinner@sydney.edu.au. Address: Level 4, Building M02C, 94 Mallet Street, Camperdown, NSW 2050, Australia. Phone: +61 450 458 201.

Supplementary appendix 1

Derivation of the equilibrium numbers of adolescents and young adults in the stocks N, V, S, and D

1.1. Derivation of the equilibrium values of N, V, S, and D

At equilibrium, the values of the stocks N, V, S, and D remain constant, i.e., their rates of change are equal to 0, so we have the equilibrium conditions

$$\frac{dN}{dt} = (1-\phi)g - uN^* - (\lambda + \mu)N^* = 0$$

$$\frac{dV}{dt} = \phi g - [1 + h(\theta - 1)]uV^* - (\lambda + \alpha\mu)V^* = 0$$

$$\frac{dS}{dt} = uN^* + [1 + h(\theta - 1)]uV^* - iS^* - (\lambda + \beta\mu)S^* = 0$$

$$\frac{dD}{dt} = iS^* - (\lambda + \gamma\mu)D^* = 0,$$

where t is time (in years), N^*, V^*, S^*, and D^* are the equilibrium values of N, V, S, and D, and all other notation is the same as in Fig. 1 of the paper. Solving the first equilibrium condition for N^* and the second equilibrium condition for V^*, we obtain $N^* = (1-\phi)g/(u + \lambda + \mu)$ and $V^* = \phi g/[u + h(\theta - 1)u + \lambda + \alpha\mu]$. Substituting these expressions for N^* and V^* into the third equilibrium condition and solving for S^* gives us the following

$$S^* = \frac{(1-\phi)g[u + h(\theta - 1)u + \lambda + \alpha\mu]u + \phi g(u + \lambda + \mu)[1 + h(\theta - 1)]u}{[u + h(\theta - 1)u + \lambda + \alpha\mu](u + \lambda + \mu)(i + \lambda + \beta\mu)},$$

which can be substituted into the fourth equilibrium condition to obtain the equilibrium number of adolescents and young adults with full-threshold mental disorders

$$D^* = \frac{i(1-\phi)g[u + h(\theta - 1)u + \lambda + \alpha\mu]u + i\phi g(u + \lambda + \mu)[1 + h(\theta - 1)]u}{[u + h(\theta - 1)u + \lambda + \alpha\mu](u + \lambda + \mu)(i + \lambda + \beta\mu)(\lambda + \gamma\mu)}.$$

1.2. Determining equilibrium stability

The stability of the equilibrium state (N^*, V^*, S^*, D^*) can be determined from the eigenvalues of the Jacobian matrix

$$J = \begin{bmatrix} \frac{\partial(dN/dt)}{\partial N} & \frac{\partial(dN/dt)}{\partial V} & \frac{\partial(dN/dt)}{\partial S} & \frac{\partial(dN/dt)}{\partial D} \\ \frac{\partial(dV/dt)}{\partial N} & \frac{\partial(dV/dt)}{\partial V} & \frac{\partial(dV/dt)}{\partial S} & \frac{\partial(dV/dt)}{\partial D} \\ \frac{\partial(dS/dt)}{\partial N} & \frac{\partial(dS/dt)}{\partial V} & \frac{\partial(dS/dt)}{\partial S} & \frac{\partial(dS/dt)}{\partial D} \\ \frac{\partial(dD/dt)}{\partial N} & \frac{\partial(dD/dt)}{\partial V} & \frac{\partial(dD/dt)}{\partial S} & \frac{\partial(dD/dt)}{\partial D} \end{bmatrix}$$

$$= \begin{bmatrix} -u-(\lambda+\mu) & 0 & 0 & 0 \\ 0 & -[1+h(\theta-1)]u-(\lambda+\alpha\mu) & 0 & 0 \\ u & [1+h(\theta-1)]u & -i-(\lambda+\beta\mu) & 0 \\ 0 & 0 & i & -(\lambda+\gamma\mu) \end{bmatrix}.$$

J is a lower triangular matrix, so its eigenvalues are equal to the diagonal elements $-u-(\lambda+\mu)$, $-[1+h(\theta-1)]u-(\lambda+\alpha\mu)$, $-i-(\lambda+\beta\mu)$, and $-(\lambda+\gamma\mu)$. As the parameters u, i, λ, μ, α, β, and γ are positive and $1+h(\theta-1)$ is equal to or greater than 1, all four eigenvalues are negative, so that (N^*, V^*, S^*, D^*) is always stable (i.e., we expected that N, V, S, and D will return to their equilibrium values when the system is disturbed).

Supplementary appendix 2

Empirical values for ϕ and θ (i.e., the proportion of adolescents turning 12 years at increased risk of mental illness and the relative risk of symptom onset, respectively)

Table S1. Empirical estimates of ϕ and θ for a diverse selection of risk-modifying factors (see panel D of Fig. 2).

Risk-modifying factor	ϕ	θ	Reference
Substantiated childhood maltreatment	0.1092	3.11	Abajobir et al. (2017)
Sexual minority	0.0636	5.43	Amos et al. (2020)
Socioeconomic disadvantage	0.2	1.6738	Barros et al. (2018)
Parental generalised anxiety disorder (GAD)	0.1016	1.93	Beesdo et al. (2010)
Parental death	0.0233	1.46	Björkenstam et al. (2017)
Parental separation	0.2469	1.58	Björkenstam et al. (2017)
Parental criminality	0.0415	1.78	Björkenstam et al. (2017)
Residential instability	0.0135	1.89	Björkenstam et al. (2017)
Household receiving public assistance	0.045	1.95	Björkenstam et al. (2017)
Parental substance abuse	0.0301	2.09	Björkenstam et al. (2017)
Parental psychiatric morbidity	0.051	2.11	Björkenstam et al. (2017)
Child welfare intervention	0.0136	2.79	Björkenstam et al. (2017)
Childhood trauma	0.188	1.2171	Copeland et al. (2018)
Male sex	0.513	1.0602	Dalsgaard et al. (2020)
Race/ethnicity - non-Hispanic black	0.1742	1.2221	Georgiades et al. (2018)
Race/ethnicity - Hispanic	0.1208	1.2272	Georgiades et al. (2018)
Parental divorce or separation	0.309	1.4253	Gilman et al. (2003)
Residential instability	0.379	1.4717	Gilman et al. (2003)
Not in education, employment, or training (NEET)	0.1157	1.68	Goldman-Mellor et al. (2016)
Not in education, employment, or training (NEET)	0.1195	1.2894	Gutiérrez-García et al. (2017)
Physical injury requiring medical care	0.1115	1.38	Jenness et al. (2017)
Hospitalisation for infection	0.31	1.84	Köhler-Forsberg et al. (2019)
Maternal bacterial infection during pregnancy	0.2269	1.8	Lee et al. (2020)
Bullying (victim)	0.262	2.14	Lereya et al. (2015)
Maternal depressive disorder	0.304	2.261	Marmorstein et al. (2004)
Parental substance abuse	0.0631	1.86	Martikainen et al. (2018)
Substantiated childhood sexual abuse	0.0143	3.36	Mills et al. (2016)
Polygenic risk score (decile 10)	0.1	1.5189	Musliner et al. (2019)
Polygenic risk score (deciles 9-10)	0.2	1.5058	Musliner et al. (2019)
Polygenic risk score (deciles 8-10)	0.3	1.5214	Musliner et al. (2019)
Polygenic risk score (deciles 7-10)	0.4	1.524	Musliner et al. (2019)
Polygenic risk score (deciles 6-10)	0.5	1.5301	Musliner et al. (2019)
Polygenic risk score (deciles 5-10)	0.6	1.5716	Musliner et al. (2019)
Polygenic risk score (deciles 4-10)	0.7	1.5737	Musliner et al. (2019)
Polygenic risk score (deciles 3-10)	0.8	1.653	Musliner et al. (2019)
Polygenic risk score (deciles 2-10)	0.9	1.8511	Musliner et al. (2019)
Parental post-traumatic stress disorder	0.146	1.75	Nielsen et al. (2019)
Temporary separation from parents	0.1335	1.18	Räikkönen et al. (2011)
Bullying (victim)	0.0642	2.1	Sourander et al. (2007)
Bullying (both bully and victim)	0.0276	4.5	Sourander et al. (2007)
Maternal antenatal depression	0.1172	1.85	Srinivasan et al. (2020)
Maternal postnatal depression	0.0751	2.09	Srinivasan et al. (2020)
Physical inactivity	0.0919	1.1964	Wu et al. (2018)

References

Abajobir, A. A., Kisely, S., Scott, J. G., Williams, G., Clavarino, A., Strathearn, L., Najman, J. M., 2017. Childhood maltreatment and young adulthood hallucinations, delusional experiences, and psychosis: a longitudinal study. Schizophr. Bull. 43, 1045−1055.

Amos, R., Manalastas, E. J., White, R., Bos, H., Patalay, P., 2020. Mental health, social adversity, and health-related outcomes in sexual minority adolescents: a contemporary national cohort study. Lancet Child Adolesc. Health 4, 36−45.

Barros, F. C., Matijasevich, A., Santos, I. S., Horta, B. L., da Silva, B. G. C., Munhoz, T. N., Fazel, S., Stein, A., Pearson, R. M., Anselmi, L., Rohde, L. A., 2018. Social inequalities in mental disorders and substance misuse in young adults. Soc. Psychiatry Psychiatr. Epidemiol. 53, 717−726.

Beesdo, K., Pine, D. S., Lieb, R., Wittchen, H., 2010. Incidence and risk patterns of anxiety and depressive disorders and categorization of generalized anxiety disorder. Arch. Gen. Psychiatry 67, 47−57.

Björkenstam, E., Vinnerljung, B., Hjern, A., 2017. Impact of childhood adversities on depression in early adulthood: a longitudinal cohort study of 478,141 individuals in Sweden. J. Affect. Disord. 223, 95−100.

Copeland, W. E., Shanahan, L., Hinesley, J., Chan, R. F., Aberg, K. A., Fairbank, J. A., van den Oord, E. J. C. G., Costello, J., 2018. Association of childhood trauma exposure with adult psychiatric disorders and functional outcomes. J. Am. Med. Assoc. Netw. Open 1, e184493.

Dalsgaard, S., Thorsteinsson, E., Trabjerg, B. B., Schullehner, J., Plana-Ripoll, O., Brikell, I., Wimberley, T., Thygesen, M., Madsen, K. B., Timmerman, A., Schendel, D., McGrath, J. J., Mortensen, P. B., Pedersen, C. B., 2020. Incidence rates and cumulative incidences of the full spectrum of diagnosed mental disorders in childhood and adolescence. J. Am. Med. Assoc. Psychiatry 77, 155−164.

Georgiades, K., Paksarian, D., Rudolph, K. E., Merikangas, K. R., 2018. Prevalence of mental disorders and service use by immigrant generation and race/ethnicity among U. S. adolescents. J. Am. Acad. Child Adolesc. Psychiatry 57, 280−287.

Gilman, S. E., Kawachi, I., Fitzmaurice, G. M., Buka, S. L., 2003. Socio-economic status, family disruption and residential stability in childhood: relation to onset, recurrence and remission of major depression. Psychol. Med. 33, 1341−1355.

Goldman-Mellor, S., Caspi, A., Arseneault, L., Ajala, N., Ambler, A., Danese, A., Fisher, H., Hucker, A., Odgers, C., Williams, T., Wong, C., Moffitt, T. E., 2016. Committed to work but vulnerable: self-perceptions and mental health in NEET 18-year olds from a contemporary British cohort. J. Child Psychol. Psychiatry 57, 196−203.

Gutiérrez-García, R. A., Benjet, C., Borges, G., Méndez Ríos, E., Medina-Mora, M. E., 2017. NEET adolescents grown up: eight-year longitudinal follow-up of education, employment and mental health from adolescence to early adulthood in Mexico City. Eur. Child Adolesc. Psychiatry 25, 1459−1469.

Jenness, J. L., Witt, C. E., Quistberg, D. A., Johnston, B. D., Rowhani-Rahbar, A., Mackelprang, J. L., McLaughlin, K. A., Vavilala, M. S., Rivara, F. P., 2017. Association of physical injury and mental health: results from the national comorbidity survey- adolescent supplement. J. Psychiatr. Res. 92, 101−107.

Köhler-Forsberg, O., Petersen, L., Gasse, C., Mortensen, P. B., Dalsgaard, S., Yolken, R. H., Mors, O., Benros, M. E., 2019. A nationwide study in Denmark of the association between treated infections and the subsequent risk of treated mental disorders in children and adolescents. J. Am. Med. Assoc. Psychiatry 76, 271−279.

Lee, Y. H., Cherkerzian, S., Seidman, L. J., Papandonatos, G. D., Savitz, D. A., Tsuang, M. T., Goldstein, J. M., Buka, S. L., 2020. Maternal bacterial infection during pregnancy and offspring risk of psychotic disorders: variation by severity of infection and offspring sex. Am. J. Psychiatry 177, 66−75.

Lereya, S. T., Copeland, W. E., Zammit, S., Wolke, D., 2015. Bully/victims: a longitudinal, population-based cohort study of their mental health. Eur. Child Adolesc. Psychiatry 24, 1461−1471.

Marmorstein, N. R., Malone, S. M., Iacono, W. G., 2004. Psychiatric disorders among offspring of depressed mothers: associations with paternal psychopathology. Am. J. Psychiatry 161, 1588−1594.

Martikainen, P., Korhonen, K., Moustgaard, H., Aaltonen, M., Remes, H., 2018. Substance abuse in parents and subsequent risk of offspring psychiatric morbidity in late adolescence and early adulthood: a longitudinal analysis of siblings and their parents. Soc. Sci. Med. 217, 106−111.

Mills, R., Kisely, S., Alati, R., Strathearn, L., Najman, J., 2016. Self-reported and agency-notified child sexual abuse in a population-based birth cohort. J. Psychiatr. Res. 74, 87−93.

Musliner, K. L., Mortensen, P. B., McGrath, J. J., Suppli, N. P., Hougaard, D. M., Bybjerg-Grauholm, J., Bækvad-Hansen, M., Andreassen, O., Pedersen, C. B., Pedersen, M. G., Mors, O., Nordentoft, M., Børglum, A. D., Werge, T., Agerbo, E., 2019. Association of polygenic liabilities for major depression, bipolar disorder, and schizophrenia with risk for depression in the Danish population. J. Am. Med. Assoc. Psychiatry 76, 516−525.

Nielsen, M. B., Carlsson, J., Rimvall, M. K., Petersen, J. H., Norredam, M., 2019. Risk of childhood psychiatric disorders in children of refugee parents with post-traumatic stress disorder: a nationwide, register-based, cohort study. Lancet Public Health 4, e353−e359.

Räikkönen, K., Lahti, M., Heinonen, K., Pesonen, A., Wahlbeck, K., Kajantie, E., Osmond, C., Barker, D. J. P., Eriksson, J. G., 2011. Risk of severe mental disorders in adults separated temporarily from their parents in childhood: the Helsinki birth cohort study. J. Psychiatr. Res. 45, 332−338.

Sourander, A., Jensen, P., Rönning, J. A., Niemelä, S., Helenius, H., Sillanmäki, L., Kumpulainen, K., Piha, J., Tamminen, T., Moilanen, I., Almqvist, F., 2007. What is the early adulthood outcome of boys who bully or are bullied in childhood? The Finnish 'From a Boy to a Man' study. Pediatrics 120, 397−404.

Srinivasan, R., Pearson, R. M., Johnson, S., Lewis, G., Lewis, G., 2020. Maternal perinatal depressive symptoms and offspring psychotic experiences at 18 years of age: a longitudinal study. Lancet Psychiatry 7, 431−440.

Wu, X., Bastian, K., Ohinmaa, A., Veugelers, P., 2018. Influence of psychical activity, sedentary behaviour, and diet quality in childhood on the incidence of internalizing and externalizing disorders during adolescence: a population-based cohort study. Ann. Epidemiol. 28, 86−94.

Supplementary appendix 3

Population-level effectiveness of multi-component prevention programs for youth mental disorders

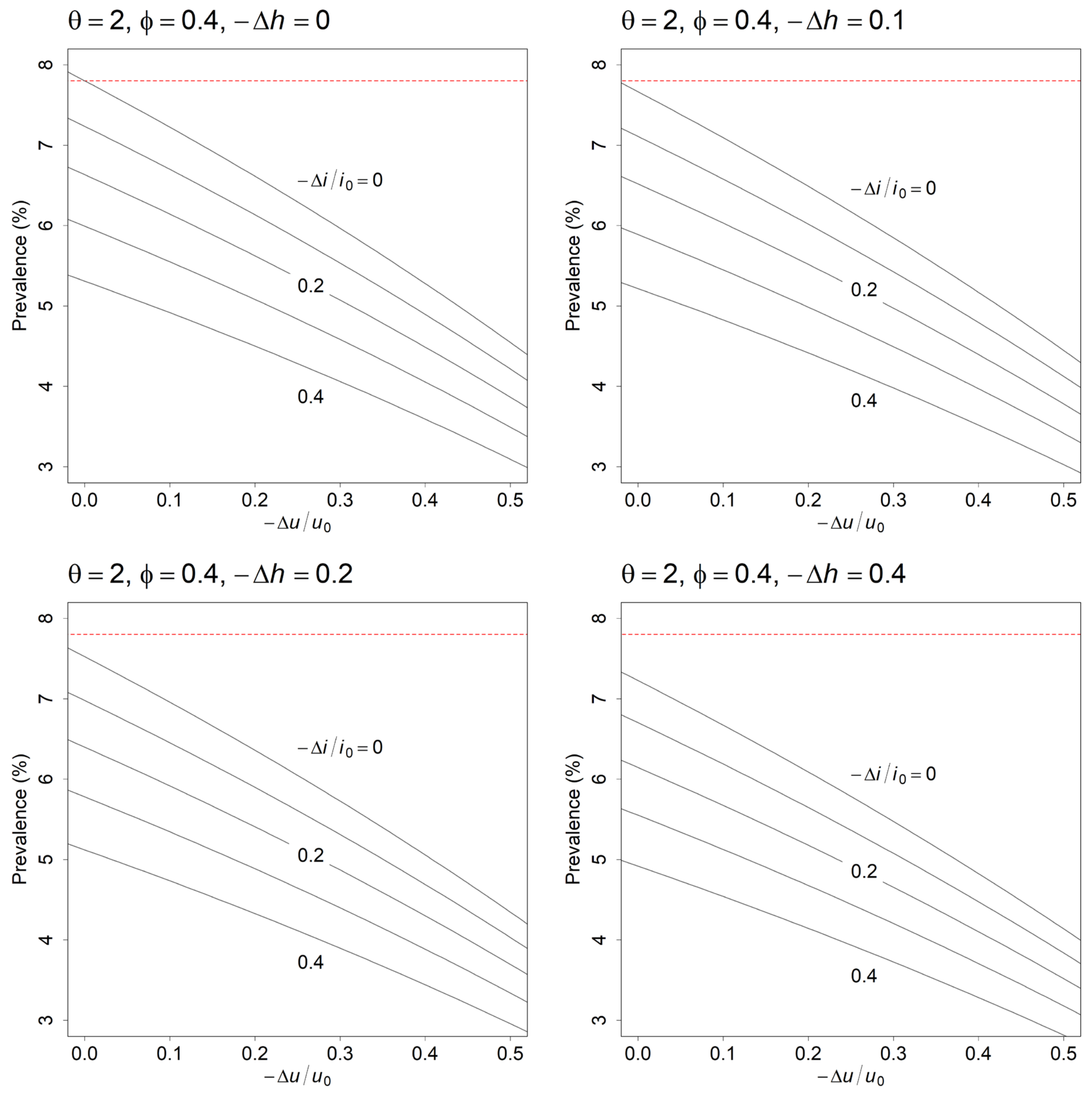

Fig. S1. Post-intervention equilibrium prevalence of full-threshold disorders for a multi-component prevention program incorporating universal, selective, and indicated interventions with varying individual-level effects ($-\Delta u/u_0$, $-\Delta h$, and $-\Delta i/i_0$, respectively). The red dashed line in the upper part of each plot corresponds to the equilibrium prevalence of full-threshold disorders prior to implementing the program (7.80% in all cases).

Supplementary appendix 4

Results of analyses using mental disorder prevalence estimates from the World Mental Health Surveys

For the analyses presented in the paper, we assumed values for the parameters u and i (i.e., the per capita rate of symptom onset for the non-vulnerable population and the per capita disease progression rate, respectively) yielding equilibrium proportions of adolescents and young adults with subthreshold symptoms and full-threshold disorders equal to empirical estimates for Australia, obtained by multiplying the prevalence of mental disorders for 16–24-year-olds in 2007 (26.4%; Australian Bureau of Statistics, 2008) by the proportions of adolescents and young adults presenting to headspace services allocated to clinical stages 1a–b and 2–4 (0.704 and 0.296, respectively; Mei et al., 2019). The extent to which our results depend on these parameter assumptions was assessed by repeating the analyses presented in Fig. 3 using disorder prevalence estimates from the World Health Organization's World Mental Health Surveys (Demyttenaere et al., 2004). Figs S2–S4 present the results of analyses for Nigeria, Japan, and the United States, respectively. In calculating u and i for each country, we equated mild–moderate mental disorders with clinical stages 1a–b (i.e., subthreshold symptoms) and severe mental disorders with clinical stages 2 and above (full-threshold disorders). Clearly, this approach is very rough; however, our aim here is to examine the impact of modifying our parameter assumptions, not to accurately estimate the prevalence of subthreshold and full-threshold symptoms. Although the three countries have widely varying values for the prevalence of severe disorders (Nigeria 0.4%, Japan 1.5%, United States, 7.7%) and the proportion of all disorders that are severe (Nigeria 8.5%, Japan 17.0%, United States, 29.3%), the results in Figs S2–S4 are qualitatively similar to one another and to those in Fig. 3 of the paper.

References

Australian Bureau of Statistics, 2008. National Survey of Mental Health and Wellbeing. Summary of results, 2007. Available at: https://www.abs.gov.au/statistics/health/mental-health/national-study-mental-health-and-wellbeing/2007.

Demyttenaere, K., Bruffaerts, R., Posada-Villa, J., Gasquet, I., Kovess, V., Lepine, J. P., Angermeyer, M. C., Bernert, S., de Girolamo, G., Morosini, P., Polidori, G., Kikkawa, T., Kawakami, N., Ono, Y., Takeshima, T., Uda, H., Karam, E. G., Fayyad, J. A., Karam, A. N., Mneimneh, Z. N., Medina-Mora, M. E., Borges, G., Lara, C., de Graaf, R., Ormel, J., Gureje, O., Shen, Y., Huang, Y., Zhang, M., Alonso, J., Haro, J. M., Vilagut, G., Bromet, E. J., Gluzman, S., Webb, C., Kessler, R. C., Merikangas, K. R., Anthony, J. C., Von Korff, M. R., Wang, P. S., Brugha, T. S., Aguilar-Gaxiola, S., Lee, S., Heeringa, S., Pennell, B., Zaslavsky, A. M., Ustun, T., B., Chatterji, S., 2004. Prevalence, severity, and unmet need for treatment of mental disorders in the World Health Organization World Mental Health Surveys. J. Am. Med. Assoc. 291, 2581−2590.

Mei, C., McGorry, P. D., Hickie, I. B., 2019. Clinical staging and its potential to enhance mental health care. In: McGorry, P. D., Hickie, I. B. (Eds.), Clinical staging in psychiatry. Cambridge University Press, Cambridge, pp. 12−33.

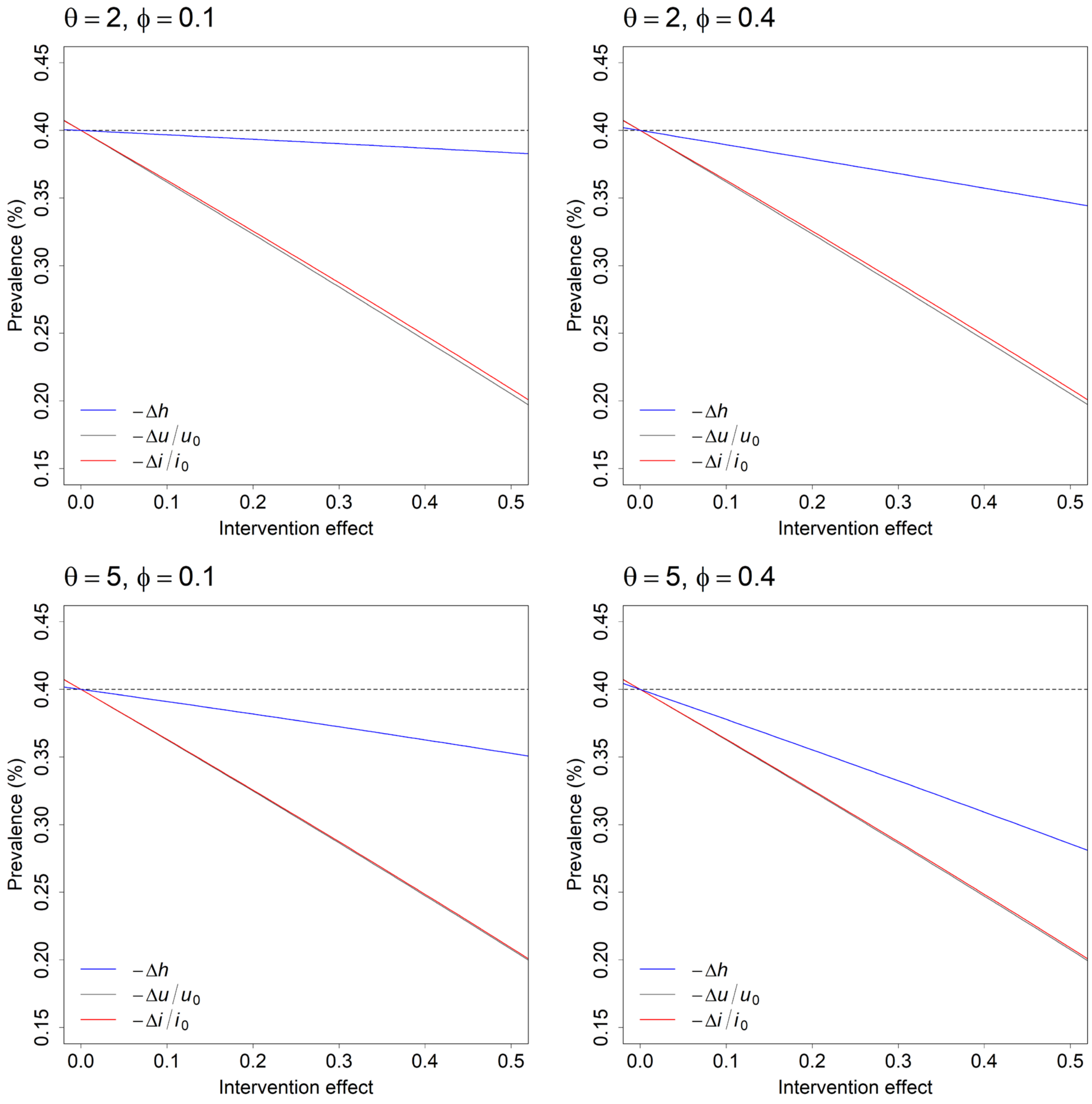

Fig. S2. Post-intervention equilibrium prevalence of mental disorders for universal (grey), selective (blue), and indicated (red) preventive interventions with varying individual-level effects (cf. Fig. 3). Pre-intervention values for u and i (u_0 and i_0, respectively) were derived from mental disorder prevalence estimates for Nigeria (Demyttenaere et al., 2004).

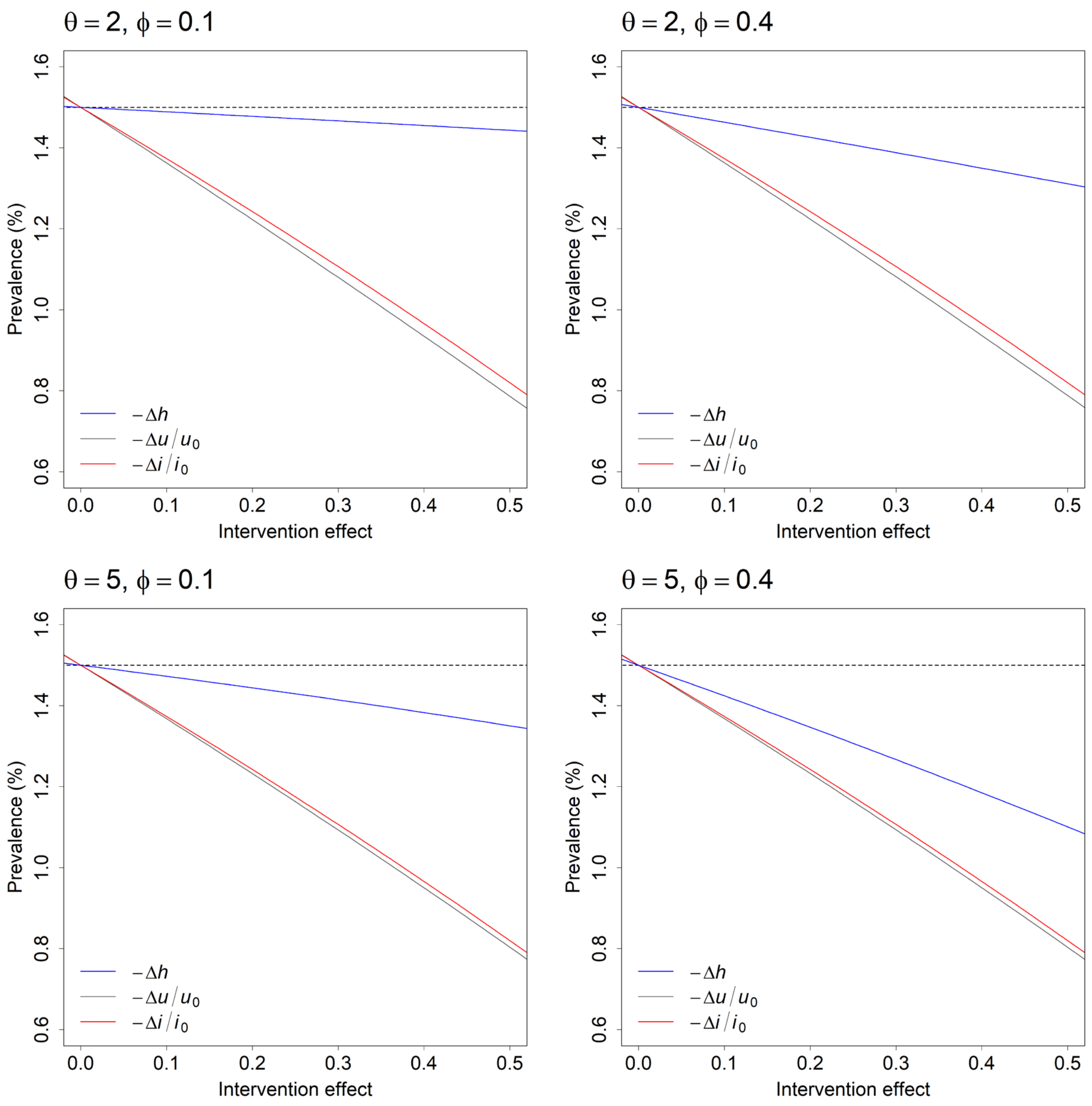

Fig. S3. Post-intervention equilibrium prevalence of mental disorders for universal (grey), selective (blue), and indicated (red) preventive interventions with varying individual-level effects (cf. Fig. 3). Pre-intervention values for u and i (u_0 and i_0, respectively) were derived from mental disorder prevalence estimates for Japan (Demyttenaere et al., 2004).

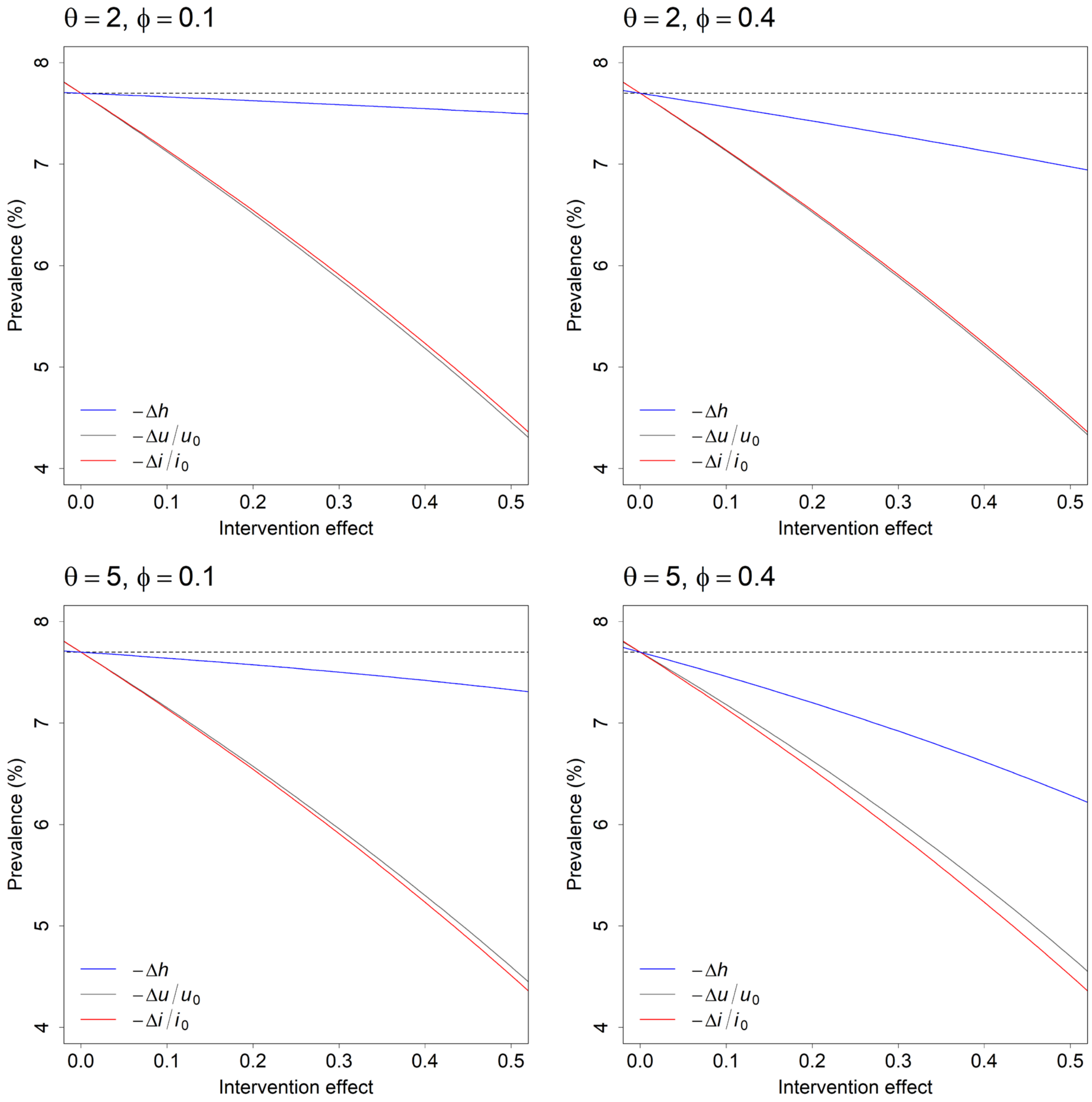

Fig. S4. Post-intervention equilibrium prevalence of mental disorders for universal (grey), selective (blue), and indicated (red) preventive interventions with varying individual-level effects (cf. Fig. 3). Pre-intervention values for u and i (i.e., u_0 and i_0) were derived from mental disorder prevalence estimates for the United States (Demyttenaere et al., 2004).

Supplementary appendix 5

Prevalence of full-threshold mental disorders over time for universal, selective, and indicated preventive interventions with identical population-level effects (at equilibrium)

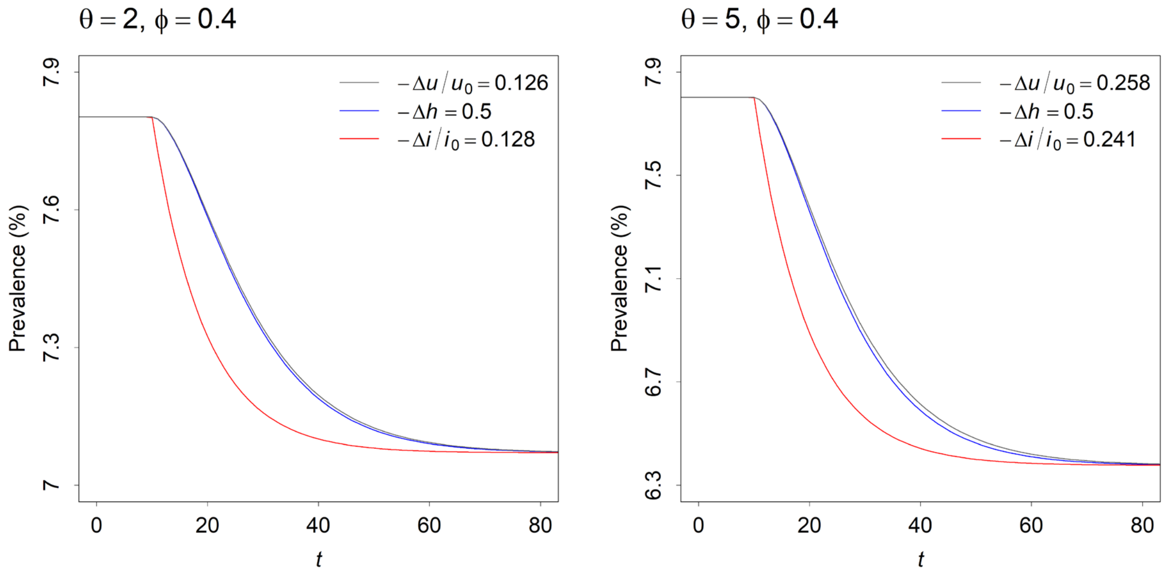

Fig. S5. Prevalence of youth mental disorders over time for hypothetical universal (grey), selective (blue), and indicated (red) preventive interventions (introduced at time t = 10) with identical population-level effects. In each plot, all three interventions yield the same post-intervention disorder prevalence at equilibrium; however, prevalence approaches the equilibrium value more rapidly for the indicated intervention than for the universal and selective interventions.
